# HTLV-1-Mediated Epigenetic Pathway to Adult T-Cell Leukemia–Lymphoma

**DOI:** 10.3389/fmicb.2018.01686

**Published:** 2018-07-24

**Authors:** Makoto Yamagishi, Dai Fujikawa, Toshiki Watanabe, Kaoru Uchimaru

**Affiliations:** ^1^Department of Computational Biology and Medical Sciences, Graduate School of Frontier Sciences, The University of Tokyo, Tokyo, Japan; ^2^The Institute of Medical Science, The University of Tokyo, Tokyo, Japan

**Keywords:** HTLV-1, ATLL, epigenetics, EZH2, gene expression, gene mutations

## Abstract

Human T-cell leukemia virus type 1 (HTLV-1), the first reported human oncogenic retrovirus, is the etiologic agent of highly aggressive, currently incurable diseases such as adult T-cell leukemia–lymphoma (ATL) and HTLV-1-associated myelopathy/tropical spastic paraparesis (HAM/TSP). HTLV-1 proteins, including Tax and HBZ, have been shown to have critical roles in HTLV-1 pathogenicity, yet the underlying mechanisms of HTLV-1-driven leukemogenesis are unclear. The frequent disruption of genetic and epigenetic gene regulation in various types of malignancy, including ATL, is evident. In this review, we illustrate a focused range of topics about the establishment of HTLV-1 memory: (1) genetic lesion in the Tax interactome pathway, (2) gene regulatory loop/switch, (3) disordered chromatin regulation, (4) epigenetic lock by the modulation of epigenetic factors, (5) the loss of gene fine-tuner microRNA, and (6) the alteration of chromatin regulation by HTLV-1 integration. We discuss the persistent influence of Tax-dependent epigenetic changes even after the disappearance of HTLV-1 gene expression due to the viral escape from the immune system, which is a remaining challenge in HTLV-1 research. The summarized evidence and conceptualized description may provide a better understanding of HTLV-1-mediated cellular transformation and the potential therapeutic strategies to combat HTLV-1-associated diseases.

## Introduction

Human T-cell leukemia virus type 1 (HTLV-1) infection (Poiesz et al., 1980; Hinuma et al., 1981; Yoshida et al., 1982) is associated with the development of adult T-cell leukemia–lymphoma (ATL) and HTLV-1-associated myelopathy/tropical spastic paraparesis (HAM/TSP), although most virus carriers remain asymptomatic throughout their lifespan. ATL is a highly aggressive T-cell malignancy refractory to the currently available combination chemotherapies (Uchiyama et al., 1977; Tsukasaki et al., 2007; Katsuya et al., 2012). HAM/TSP, a debilitating neuro-inflammatory disease, expresses chronic spinal cord inflammation and progressive myelopathic symptoms (Gessain et al., 1985; Osame et al., 1986).

Accumulating evidence has shown that HTLV-1 exhibits complicated involvement in the pathogenesis (Matsuoka and Jeang, 2007; Yamagishi and Watanabe, 2012). In particular, HTLV-1 Tax significantly affects host gene expression and interacts with multiple partner proteins (Boxus et al., 2008; Chevalier et al., 2012; Simonis et al., 2012). Moreover, Tax-transgenic mice develop malignant lymphoma, suggesting that Tax is an oncoprotein (Hasegawa et al., 2006; Ohsugi et al., 2007). The evolution of viral genes with virus expansion indicates that leukemogenesis by Tax is selectively advantageous for viral replication and cell proliferation. Transgenic expression of *HBZ* in CD4^+^ T-cells also induces T-cell lymphomas and systemic inflammation in mice (Satou et al., 2011). Tax and HBZ certainly contribute to leukemogenesis in HTLV-1-infected T-cells. However, considering the low rate of incidence, clinical observation implies that HTLV-1 lacks a strong capacity to induce leukemogenesis, in contrast to other animal leukemia viruses.

Notably, most leukemic cells do not express viral genes, excluding HBZ (Gaudray et al., 2002; Taniguchi et al., 2005; Satou et al., 2006). Tax, a highly immunogenic protein, is not expressed in most aggressive-type ATL cases because HTLV-1 provirus is substantially silenced by proviral defect and/or an epigenetic mechanism (Tamiya et al., 1996; Koiwa et al., 2002; Taniguchi et al., 2005). It is assumed that this is one of the strategies that viruses use to evade host immune defense.

**FIGURE 1:**
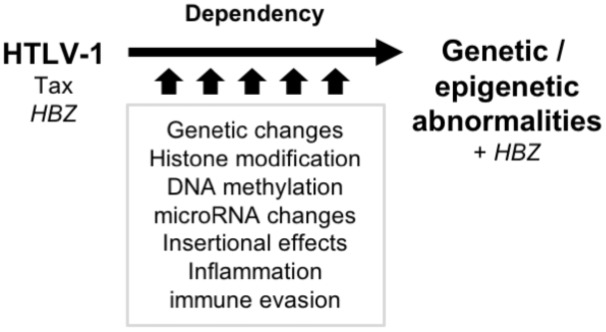
Transition of the molecular characteristics during latent period. The acquired cellular characteristics such as promoting cell proliferation and apoptotic resistance are conferred by viral genes in early-phase infected cells and by genetic/epigenetic abnormalities in late-phase ATL cells. The aberrant characteristics are acquired and imprinted, nevertheless Tax disappears. The consequent genotype and epigenotype support the differential phenotypes and the disease entities.

However, leukemic cells possess similar traits as Tax-expressing cells (Yamagishi and Watanabe, 2012). Although the reason for this seemingly paradoxical observation is yet to be determined, it is suggested that the acquired cellular characteristics, including promoting cell proliferation and apoptotic resistance, is conferred by viral genes in early-phase infected cells and by genetic/epigenetic abnormalities in late-phase, highly malignant ATL cells (**Figure 1**). Although Tax has already disappeared at the time of ATL onset, Tax and its interactome (described in later chapters) have already left multiple genetic and epigenetic memories, contributing ATL onset. This switch during leukemogenesis is indeed supported by transcriptome data; the changes in gene expression in infected cells are dominated by disordered homeostasis and the characteristics of ATL.

Although cancer is typically considered to be a genetic disease, chromatin and epigenetic aberrations as well as active roles of HBZ play important roles in tumor potentiation, initiation, and progression in ATL and HTLV-1-associated diseases. Based on recent findings, we introduce a hypothesis with important implications that might explain the underlying mechanism of the issue: the molecular memories inherited from HTLV-1.

## Transcriptome Abnormality in ATL

Cellular characteristics (i.e., phenotype) are strictly defined by the regulation of gene expression. HTLV-1 Tax directly affects host gene expression through multiple mechanisms, including the binding with host transcription factors and the perturbation of multiple signaling pathways (Ballard et al., 1988; Ruben et al., 1988; Kim et al., 1990; Migone et al., 1995; Good et al., 1996; Takemoto et al., 1997; Boxus et al., 2008). Intriguingly, the molecular hallmarks of aggressive ATL cells at the final stage of progression still comprise pronounced dysregulation of the signaling pathways that control the cell cycle, the resistance to apoptosis, and the proliferation of leukemic cells without Tax expression.

Cell cycle regulation is a typical example of the correlation between gene expression and phenotypic changes. The oncogenic function of Tax was first demonstrated in a study of cell cycle regulation. Tax inhibits cyclin-dependent kinase (CDK) inhibitor, *CDKN2A* (p16^INK4A^), via physical interaction (Suzuki et al., 1996). The mitogenic activity of Tax is exerted through the stimulation of G_1_-to-S-phase transition. Additionally, Tax affects a cohort of cell cycle-related proteins, including *CDKs, CDKN1A, CDKN1B*, and *CDKN2A*, via the regulation of their expression or physical interaction (Akagi et al., 1996; Neuveut et al., 1998; Schmitt et al., 1998; Santiago et al., 1999; Suzuki et al., 1999; de La Fuente et al., 2000; Iwanaga et al., 2001; Haller et al., 2002; Liang et al., 2002).

Comprehensive gene expression profiling revealed that several positive regulators of the cell cycle process are overexpressed in acute-type ATL, in most of which HTLV-1 sense-transcripts and the virus replication is silenced. Diverse abnormalities were also found in each of these comprehensive studies; however, several gene alterations and other critical events have been commonly implicated as the determinants of gene expression pattern. The abnormalities in the expression of different cytokines, their receptors, and various proteins that act as anti-apoptotic factors or proliferating agents are the cellular hallmarks responsible for malignant phenotypes (Tsukasaki et al., 2004; Sasaki et al., 2005; Watanabe et al., 2010; Yamagishi et al., 2012). These notable traits in the transcriptome may be genetically and epigenetically established during long-term latency periods.

## Establishment of HTLV-1 Memory

Genetic, metabolic, and environmental stimuli can induce overly restrictive or permissive epigenetic landscapes that contribute to the pathogenesis of cancer and other diseases. The restrictive chromatin states prevent the appropriate expression of tumor suppressors or block differentiation. In contrast, the permissive states allow the stochastic activation of oncogenic genes and stochastic silencing of tumor suppressor genes. The abnormal restriction or plasticity may also affect other processes mediated through factors such as chromatin–DNA repair and telomere maintenance.

Chromatin homeostasis, a basis of molecular memory (Flavahan et al., 2017), is disrupted by genetic and epigenetic stimuli (e.g., inflammation, aging, hypoxia, cell stress, developmental cues, metabolism, and pathogens). The heritable, selective adaptive changes are the hallmarks of cancers. Herein, we introduce the abnormality contributing to the molecular pathogenesis of HTLV-1 infection by tracing the function(s) of Tax and the characteristics of ATL cells.

### Genetic Lesion in Tax Interactome Pathway

Tax directly participates in genetic damage (Jeang et al., 1990; Saggioro et al., 1994; Kao and Marriott, 1999; Haoudi et al., 2003). In parallel with this, persistent proliferation, which is boosted by cell cycle progression, may cause genetic instability and create stochastic genetic lesions; ≥ 1 lesions may then act as “drivers,” allowing clonal evolution.

The recent advanced technology-based comprehensive characterization of genetic abnormalities delineated the spectrum of genetic alterations in ATL (Kataoka et al., 2015). Genomic data from a total of 426 patients with ATL identified 6,404 mainly age-related somatic mutations (2.3 mutations/Mb/sample) by whole-exome sequencing, including 6,096 single-nucleotide variants and 308 insertions–deletions, strongly suggesting that the clonal expansion of aggressive ATL cells is driven by multiple genetic abnormalities. One of the remarkable indications is that some of the somatically altered genes in ATL (mutation and copy number variation) encode the pivotal molecules that Tax physically interacts with and/or deregulates, including the components of TCR–NF-κB pathway [activated by Tax (Yamaoka et al., 1998; reviewed in Sun and Yamaoka, 2005)] and p53 and p16 tumor suppressors [inactivated by Tax (Suzuki et al., 1996; Grassmann et al., 2005)]; this strongly suggests that ATL cells still depend on the dysregulated Tax interactome even after the disappearance of Tax expression in most ATL cases, i.e., the influence of Tax is genetically imprinted in ATL cells.

### Gene Regulatory Loop/Switch

Depending on the cellular status, a transient cue such as an inflammatory cytokine can induce stable malignant transformation through a positive feedback network that is normally held in check by a host defense mechanism (Iliopoulos et al., 2009; Barabási et al., 2011; Yosef and Regev, 2011). In this manner, network motifs, including a coherent feedforward, mutual negative feedback, and positive feedback loops, may switch the cell fate in some cases.

Tax can activate several signaling pathways and lead to an abnormal gene expression pattern. For instance, it can activate NF-κB and NFAT pathways responsible for the predominant expression of IL-2 and its receptor IL2R (Ballard et al., 1988; Ruben et al., 1988; Hoyos et al., 1989; McGuire et al., 1993; Good et al., 1996), whose activation leads to a positive feedback loop. The target transcriptome of NF-κB pathway includes the genes encoding the members of the Rel family, p100/p105, NF-κB-inducing kinase (NIK), and several cytokines that stimulate the same pathway.

Negative regulators within the network are critical for the homeostasis of the regulatory motif. In the developmental process of HTLV-1-infected cells, some NF-κB negative regulators are diminished or inactivated, leading to chronic activation of the signaling pathway. For example, miR-31, a new class of negative regulator of the non-canonical NF-κB pathway, acts by regulating NIK. One mechanism of NF-κB activation without Tax is the epigenetic silencing of miR-31 in HTLV-1-infected cells and aggressive ATL cells (Yamagishi et al., 2012).

Another NF-κB negative regulator, p47, which is essential for Golgi membrane fusion, associates with the NEMO subunit of IκB kinase (IKK) complex upon TNF-α or IL-1 stimulation and inhibits IKK activation. Tax inhibits the interaction between p47 and the IKK complex. In contrast, a significant reduction of p47 expression has been reported in ATL cells, which show a high-level constitutive NF-κB activation that protects ATL cells from apoptosis in a Tax-independent manner (Shibata et al., 2012). These findings indicate that defenseless signaling may cause automatically and chronically activated signaling pathways (Yamagishi et al., 2015), possibly even after the loss of Tax.

### Chromatin Regulation

Chromatin is the fundamental medium through which transcription factors, signaling pathways, and various other cues influence gene activity. A dynamic change of the chromatin conformation reinforces regulatory activity or repression at each locus and causes reorganization in response to appropriate intrinsic and extrinsic stimuli.

Genes encoding epigenetic factors, including SWI/SNF complex members and DNA methylation modifiers, are among the most frequently mutated genes in human cancers (Lawrence et al., 2014). However, the genetic changes of such epigenetic factors are less common in ATL, although epigenetic dysregulation such as DNA methylation and histone acetylation is observed at each investigated locus (Nosaka et al., 2000; Tsuchiya et al., 2000; Hofmann et al., 2001; Yasunaga et al., 2004; Yoshida et al., 2004; Yang et al., 2005; Daibata et al., 2007; Taniguchi et al., 2008).

The ATL is also characterized by prominent CpG island DNA hypermethylation, leading to transcriptional silencing (Kataoka et al., 2015). Approximately 40% of the cases showed the CpG island methylator phenotype without any mutation at *TET2, IDH2*, and *DNMT3A*. Additionally, C2H2-type zinc finger genes (implicated in the suppression of endogenous and exogenous retroviruses) were hypermethylated and silenced. Furthermore, the hypermethylation of MHC-I expression may contribute to immune evasion.

When we consider the chromatin aberrations that confer plasticity, the polycomb family and its substrate histone, H3K27, are of particular interest. EZH2 can repress a wide range of genes by catalyzing the trimethylation of H3K27 (H3K27me3). Regarding the cellular function, EZH2 and H3K27me3 act in a highly context-dependent manner. EZH2 gain-of-function mutations may be oncogenic in a B-cell lineage (Morin et al., 2010; Yap et al., 2011). In addition, an aberrant activation of polycomb repressive complex 2 (PRC2) mainly based on the overexpression of EZH2 is frequently observed in hematological malignancies and solid tumors (Yamagishi and Uchimaru, 2017). In contrast, EZH2 is genetically inactivated in myelodysplastic syndromes (Ernst et al., 2010) and T-cell acute lymphoblastic leukemia (Ntziachristos et al., 2012).

We recently analyzed the pattern of ATL histone modification and integrated it with the transcriptome from primary ATL cells to decipher the ATL-specific “epigenetic code” (Kobayashi et al., 2014; Fujikawa et al., 2016). PRC2-mediated H3K27me3 is significantly and frequently reprogrammed at half of genes in ATL cells. A large proportion of abnormal gene downregulation is observed at an early stage of disease progression, which is explained by H3K27me3 accumulation. Global H3K27me3 alterations involve ATL-specific gene expression changes that include several tumor suppressors, transcription factors, epigenetic modifiers, miRNAs, and developmental genes (Fujikawa et al., 2016), suggesting the diverse outcomes of the PRC2-dependent hierarchical regulation.

Importantly, the Tax-dependent immortalized cells also show significantly similar H3K27me3 reprogramming as that of ATL cells. A majority of the epigenetic silencing occurs in leukemic cells from indolent ATL and in HTLV-1-infected premalignant T-cells from asymptomatic HTLV-1 carriers.

The important implications for deciphering the triggers of the specific histone code are physical interaction and other influences on the host epigenetic machinery by Tax, including the key histone modifiers HDAC1 (Ego et al., 2002), SUV39H1 (Kamoi et al., 2006), SMYD3 (Yamamoto et al., 2011), and EZH2 (Fujikawa et al., 2016).

### Epigenetic Lock by Modulation of Epigenetic Factors

The functional classification of genes has revealed that genes epigenetically suppressed by H3K27me3 are enriched in certain biological processes, including transcriptional regulation and histone modifiers, in ATL. Among these, the expression of *KDM6B*, encoding a JMJD3 demethylase of H3K27me3, is significantly downregulated upon H3K27me3 gain (Fujikawa et al., 2016). Because JMJD3 downregulation causes the global accumulation of H3K27me3, ATL cells seemingly acquire a coherent pattern that produces and maintains the systematic abnormality of H3K27me3.

Another coherent pattern is observed in EZH2 regulation. EZH2 is sensitive to promiscuous signaling networks, including NF-κB pathway. Upregulated EZH2 causes excessive PRC2 activity and suppresses multiple target genes such as NF-κB negative regulators (Yamagishi et al., 2012); this forms a positive feedback loop. HTLV-1 Tax is significantly involved in this motif by interacting with EZH2 and activating NF-κB pathway (Fujikawa et al., 2016). Regarding the chronic activation of PRC2 without Tax, an initial triggering event is unnecessary for the maintenance of epigenetic loop.

### Loss of Gene Fine-Tuner microRNA

Among the regulators of gene expression, microRNAs are recognized as “buffers” and/or “fine-tuners.” MicroRNA can reduce the noise in gene expression; thus, the loss of microRNA may create perturbed gene expression at the post-transcriptional level (Huntzinger and Izaurralde, 2011; Ebert and Sharp, 2012).

One of the key characteristics of ATL is the global downregulation of microRNA (Yamagishi et al., 2012). Although it has not been experimentally demonstrated, the loss of functional small RNA may cause disordered gene expression through transcriptional and post-transcriptional levels. Notably, this global loss is caused and imprinted by HTLV-1-induced H3K27me3 accumulation, suggesting that the global loss of microRNA is one of the processes required for the developmental pathway leading to ATL.

### Alteration of Chromatin Regulation by HTLV-1 Integration

Recent advances regarding insertional effects by HTLV-1 have provided critical implications. Satou et al. (2016) found that CTCF (a key regulator of chromatin structure and function) binds to the provirus in the provirus pX region and acts as an enhancer blocker, leading to long-distance interactions with flanking host chromatin. Indeed, HTLV-1 was reported to alter local higher-order chromatin structure and gene expression in the host genome (Satou et al., 2016).

Rosewick et al. (2017) employed stranded RNA-seq data in combination with improved DNA-seq-based high-throughput mapping of integration sites and found that HTLV-1/BLV proviruses are integrated near cancer drivers, which they affect via provirus-dependent transcription termination or as a result of viral antisense RNA-dependent *cis*-perturbation. Remarkably, a similar result was observed at polyclonal non-malignant stages, indicating that provirus-dependent host gene perturbation contributes to the initial selection of the multiple clones characterizing the asymptomatic stage, requiring additional alterations in the clone that will evolve into aggressive leukemia/lymphoma. Although the hotspots of proviral integration sites and the influence of their insertion into the host genome/epigenome are still being discussed, the previously unrecognized mechanisms may be complementary to viral gene products and the acquisition of somatic alterations in the host genome.

## Epigenetic Landscape of HTLV-1-Infected Cells

The biologist Conrad Waddington first conceptualized developmental fate decision as an epigenetic landscape wherein differentiating cells proceed downhill along the branching canals separated by the walls that restrict cell identity (Waddington, 1957).

Decades of research have revealed that transcription factors are the predominant specifiers of cellular identity (Zaret and Mango, 2016; Bradner et al., 2017). However, the topography of this “hill” seems to be determined by the chromatin pattern, which is directly regulated by epigenetic mechanisms in response to the intrinsic and extrinsic (environmental) stimuli exemplified in this review.

**FIGURE 2:**
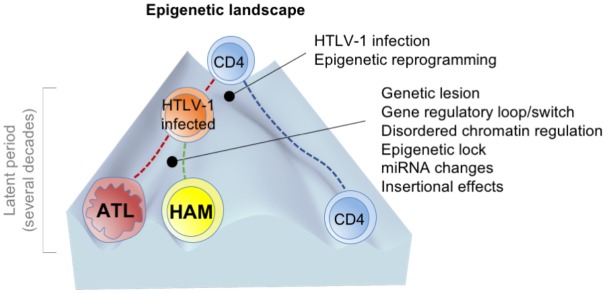
A hypothetical model of developmental pathway in HTLV-1-associated diseases [adapted from Waddington (1957)]. The height of the walls between the valleys (or canals) is determined by several molecular events. The first HTLV-1 infection and the accompanied epigenetic alterations change the cell fate. The permissive state induced by HTLV-1-infection allows following stochastic perturbations such as genetic mutations and dysregulation of the signaling pathways and clonal selection, paralleled by a decrease in transcriptional noise, and the stabilization of cell states (deepening of the valleys).

Therefore, as a hypothesis, we propose, in agreement with the established developmental pathway of HTLV-1-infected cells, that disease progression fits with the epigenetic landscape, wherein the height of the walls between the canals is determined by several molecular events (**Figure 2**).

The initial trigger for restricting gene expression is HTLV-1 infection. This violent event significantly affects cell fate, primarily by Tax and HBZ. Then, the immortalized cells possibly undergo several molecular events, as described above (including genetic and epigenetic alterations). During a long period, several aberrant characteristics are acquired and fixed, nevertheless Tax disappears. The consequent genotype and epigenotype support the differential phenotypes and the disease entities of ATL and HAM/TSP.

HTLV-1 provirus is frequently defective or silenced in ATL. However, the lesions recurrently detected in ATL cells imitate the function of Tax and would be stably inherited in the progeny of the malignant cells. This raises several critical possibilities such as that the active imprinting of the viral function into the host genome and epigenome is one of the critical steps of leukemogenesis. Furthermore, the features of ATL cells are not accidental but are the products of HTLV-1 infection. In addition to the sustained roles of HBZ (reviewed in Ma et al., 2016), some crucial outcomes (including gene mutations in the components of TCR–NF-κB pathway and abnormal H3K27me3 accumulation) and many other stochastic events shape ATL cells and their characteristics.

## Future Direction

At present, researchers and hematologists are sharing their findings on the characteristics of ATL cells. Additionally, the phenotypic characteristics of HAM/TSP have been studied. Considering the therapeutics for the HTLV-1-associated diseases and the need to eliminate the premalignant cell population, the establishment of a precise understanding of disease developmental pathways (routes, branch points, and the events that influence the landscape, as shown in **Figure 2**) is an urgent requirement. Therefore, there is a need to investigate the abnormalities contributing to the molecular pathogenesis, including those in master transcription factors and chromatin regulators. Furthermore, in addition to cellular traits, environmental parameters such as aging, cellular stress, and immune response should be integrated into our model of this process. The order of the molecular events is just a pathway of disease development. HTLV-1 infection and following epigenetic reprogramming may be an initial step of fate changes.

**FIGURE 3:**
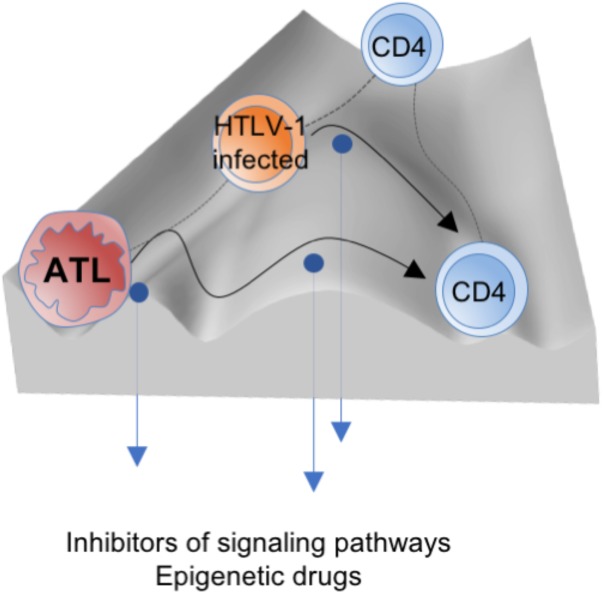
The mechanism-based medicines such as epigenetic drugs and inhibitors of signaling pathways could reprogram the fate of HTLV-1-infected cells (conceptualized as a reduction or elevation of the walls (blue arrows), which promote crossing or bypassing within the epigenetic landscape (black curved arrows) into the normal state).

Intentional regulation such as by inhibitor treatment will reprogram the fate of HTLV-1-infected cells, which can be conceptualized as a reduction or elevation of the walls between the canals in the epigenetic landscape, in line with the analogy mentioned above (**Figure 3**). Realizing the potential of such mechanism-based medicines and advanced diagnostic tools for the detection and evaluation of tumor stage and heterogeneity will require a deeper understanding of epigenetic plasticity and restriction. The road ahead is long but must be challenged to capture this major component of HTLV-1 biology and its associated diseases.

## Author Contributions

MY conceived and supervised the project, summarized and conceptualized the evidence, and wrote the paper. DF, TW, and KU discussed the new concept.

## Conflict of Interest Statement

The authors declare that the research was conducted in the absence of any commercial or financial relationships that could be construed as a potential conflict of interest.
